# Desfechos funcionais da descompressão cirúrgica para o tratamento da síndrome do túnel do tarso

**DOI:** 10.1055/s-0046-1819578

**Published:** 2026-04-17

**Authors:** Marco Antonio Casares Tamayo, Jonathan Antonio Casares Castellanos, Francisco Endara Urresta, Carlos Peñaherrera Carrillo, Alejandro Barros Castro

**Affiliations:** 1 Departamento de Ortopedia e Traumatologia, Hospital Metropolitano, Quito, Equador; 2 Pontificia Universidad Católica del Ecuador, Quito, Equador; 3 Clínica Arthros, Quito, Equador; 4 Instituto Nacional de Rehabilitación Luis Guillermo Ibarra Ibarra (LGII), Universidad Autónoma de México, Cidade do México, México; 5 Departamento de Ortopedia e Traumatologia, Universidad Internacional del Ecuador, Quito, Equador

**Keywords:** descompressão, eletromiografia, síndrome do túnel do tarso, ultrassonografia, decompression, electromyography, tarsal tunnel syndrome, ultrasonography

## Abstract

**Objetivos:**

Avaliar os desfechos funcionais da descompressão cirúrgica para tratamento da síndrome do túnel do tarso (STT) e explorar a relevância clínica de exames diagnósticos como eletromiografia (EMG), ressonância magnética (RM) e ultrassonografia (US).

**Métodos:**

Realizamos um estudo retrospectivo unicêntrico com 15 pacientes com diagnóstico clínico de STT submetidos à descompressão cirúrgica aberta (2015–2022). O tratamento conservador falhou em todos, sendo acompanhados por pelo menos 12 meses. A avaliação pré-operatória combinou o exame clínico com pelo menos um exame confirmatório (EMG, RM ou US). Os desfechos incluíram o escore (primário) da American Orthopaedic Foot and Ankle Society (AOFAS) para tornozelo e retropé, dor avaliada pela Escala Visual Analógica (EVA), diferença mínima clinicamente importante (DMCI) e complicações. A análise utilizou testes t pareados, d de Cohen e análise de covariância (ANCOVA), com ajuste conforme o escore AOFAS basal, idade, duração dos sintomas e resultados da EMG.

**Resultados:**

A idade média foi de 50,4 ± 15,6 anos; e 53,3% dos pacientes eram do sexo feminino. A duração média dos sintomas foi de 14,7 ± 7,2 meses. A EMG foi positiva em 66,7% dos casos, a RM em 60,0%, e a US em 53,3%. O escore AOFAS melhorou de 36,6 ± 7,1 para 78,1 ± 19,9 em 12 meses ( *p* < 0,001; d = 2,26) e a EVA diminuiu de 7,0 ± 0,6 para 3,4 ± 1,3 ( *p* < 0,001; d = −3,72). A DMCI foi alcançada em 80% dos casos para o escore AOFAS e em 100% para EVA. Ocorreram duas complicações menores (13,3%); não houve necessidade de nova intervenção cirúrgica. A ANCOVA sugeriu tendências de gravidade basal e cronicidade, mas sem significância estatística.

**Conclusão:**

A descompressão cirúrgica é um tratamento seguro e eficaz para a STT, proporcionando alívio significativo da dor e melhora funcional com baixas taxas de complicações. A intervenção precoce tende a gerar desfechos melhores, o que justifica a realização de mais estudos prospectivos.

## Abstract (en)

**Objectives:**

To evaluate the functional outcomes of surgical decompression for tarsal tunnel syndrome (TTS) and to explore the clinical relevance of diagnostic tests such as electromyography (EMG), magnetic resonance imaging (MRI), and ultrasound.

**Methods:**

We performed a retrospective single-center study of 15 patients with clinically diagnosed TTS who underwent open decompression (2015–2022). All had failed conservative management and completed ≥ 12 months of follow-up. Preoperative evaluation combined clinical exams with at least one confirmatory test (EMG, MRI, or ultrasound). Outcomes included the American Orthopaedic Foot and Ankle Society's (AOFAS) ankle–hindfoot score (primary), Visual Analogue Scale (VAS) pain, minimal clinically important difference (MCID), and complications. The analysis used paired *t* -tests, Cohen's d, and analysis of covariance (ANCOVA), adjusting for baseline AOFAS, age, symptom duration, and EMG results.

**Results:**

The mean age was 50.4 ± 15.6 years; and 53.3% of the patients were female. Symptom duration averaged 14.7 ± 7.2 months. The EMG scans were positive in 66.7%, MRI in 60.0%, and ultrasound in 53.3%. The AOFAS score improved from 36.6 ± 7.1 to 78.1 ± 19.9 at 12 months ( *p* < 0.001; d = 2.26), and VAS decreased from 7.0 ± 0.6 to 3.4 ± 1.3 ( *p* < 0.001; d = −3.72). The MCID was achieved in 80% for AOFAS and 100% for VAS. Two minor complications (13.3%) occurred, without need for reoperations. The ANCOVA suggested trends for baseline severity and chronicity without significance.

**Conclusion:**

Surgical decompression is a safe and effective treatment for TTS, providing significant pain relief and functional improvement with low complication rates. Early intervention shows a trend toward better outcomes, warranting further prospective studies.

## Introdução

A síndrome do túnel do tarso (STT) é uma neuropatia compressiva do nervo tibial posterior ou de seus ramos, localizados abaixo do retináculo flexor na face medial do tornozelo. Sua etiologia é multifatorial, abrangendo causas extrínsecas, como trauma, deformidade do retropé ou varizes, bem como fatores intrínsecos, incluindo cistos sinoviais, gânglios, hipertrofia muscular ou alterações fibróticas. Clinicamente, a apresentação típica é de dor em queimação no retropé e na planta do pé, parestesias e, em casos mais avançados, fraqueza ou atrofia muscular intrínseca. O impacto funcional da STT é considerável, uma vez que limita a deambulação prolongada, induz intolerância à posição ortostática e reduz a qualidade de vida relacionada à saúde, acometendo tanto indivíduos jovens e ativos quanto idosos. 1 2 3

O diagnóstico da STT é complexo. O exame clínico pode revelar um sinal de Tinel positivo sobre o retináculo flexor ou reproduzir os sintomas com manobras provocativas; no entanto, a sensibilidade e a especificidade dessas técnicas são inconsistentes. Exames complementares são frequentemente empregados para objetivar a presença de compressão nervosa, embora seu desempenho diagnóstico varie bastante.

A eletromiografia (EMG) é a ferramenta mais utilizada para documentar o comprometimento neurofisiológico, mas sua sensibilidade varia de 45 a 80%, e resultados falso-negativos são particularmente comuns nos primeiros estágios da doença. A ressonância magnética (RM) pode identificar causas secundárias, como lesões expansivas ou variantes anatômicas, mas nem sempre captura a compressão dinâmica. A ultrassonografia (US) musculoesquelética é cada vez mais utilizada por permitir a visualização em tempo real do nervo tibial e estruturas adjacentes e, assim, a avaliação dinâmica; contudo, sua acurácia diagnóstica é dependente do operador. Essa heterogeneidade entre as modalidades diagnósticas contribui para incertezas e, com frequência, atrasa a indicação cirúrgica. 4 5 6

As estratégias de tratamento conservadoras, incluindo órteses, injeções de corticosteroides e fisioterapia, podem proporcionar alívio sintomático temporário. No entanto, em pacientes com sintomas persistentes e limitação funcional, a descompressão cirúrgica do túnel do tarso ainda é o tratamento de escolha. Séries de casos relataram taxas de sucesso clínico entre 70 e 90%, com melhora significativa da dor e da função durante o acompanhamento de médio prazo. Porém, a heterogeneidade nos critérios diagnósticos, na técnica cirúrgica e nas medidas de desfecho dificulta a comparação entre estudos e pouco se sabe sobre os fatores relacionados ao paciente e à doença que modulam os resultados cirúrgicos. 7

Nesse contexto, o presente estudo visa avaliar sistematicamente os desfechos funcionais da descompressão cirúrgica para tratamento da STT em uma coorte consecutiva de pacientes, utilizando o escore da American Orthopaedic Foot and Ankle Society (AOFAS) como desfecho primário. Os desfechos secundários incluem a redução da dor medida pela Escala Visual Analógica (EVA), a obtenção da diferença mínima clinicamente importante (DMCI) e as taxas de complicações. Além disso, buscamos explorar o rendimento diagnóstico relativo de EMG, RM e US para fornecer evidências práticas sobre sua utilidade na tomada de decisões clínicas.

Para tanto, o estudo teve dois objetivos principais: avaliar os resultados funcionais (AOFAS, EVA, DMCI, complicações) após a descompressão cirúrgica para tratamento da STT; e explorar a relevância clínica de exames diagnósticos complementares (EMG, RM, US) no suporte ao diagnóstico e na orientação da indicação cirúrgica.

## Métodos

Esta pesquisa foi concebida como um estudo unicêntrico de coorte retrospectiva e realizado em uma instituição terciária de referência com uma unidade especializada em pé e tornozelo. O protocolo do estudo foi conduzido de acordo com os padrões éticos institucionais, em conformidade com os princípios da Declaração de Helsinque.

A população do estudo consistiu em 15 pacientes consecutivos diagnosticados com STT que foram submetidos à descompressão cirúrgica entre janeiro de 2015 e dezembro de 2022. Os critérios de inclusão foram adultos maiores de 18 anos com diagnóstico clínico de STT caracterizada por dor na região medial do tornozelo ou na planta do pé, parestesias ou déficit neurológico compatível com compressão do nervo tibial e que não responderam ao tratamento conservador por pelo menos 3 meses. O tratamento conservador incluiu modificação da atividade, repouso e uso de anti-inflamatórios não esteroidais, além de fisioterapia diária com ênfase em exercícios de alongamento e deslizamento neural, que podem melhorar a flexibilidade e minimizar a compressão.

Os pacientes deveriam apresentar dados clínicos completos, tanto à primeira avaliação quanto ao acompanhamento, incluindo escores padronizados de desfecho. Os critérios de exclusão incluíram cirurgia prévia no retropé, neuropatias sistêmicas (ex.: polineuropatia diabética), artropatias inflamatórias ou prontuários clínicos incompletos.

Todos os pacientes foram submetidos a uma avaliação pré-operatória uniforme, composta por exame clínico detalhado e pelo menos um exame complementar confirmatório. A avaliação clínica incluiu inspeção, palpação ao longo do retináculo flexor e manobras provocativas, como o sinal de Tinel ou o teste de dorsiflexão-eversão. Além disso, a EMG foi utilizada em todos os pacientes para documentar o comprometimento neurofisiológico do nervo tibial e foi positivo em dez casos. Em seguida, a US musculoesquelética foi realizada em dez pacientes com sintomas ambíguos para avaliação dinâmica do nervo tibial e das estruturas adjacentes, como dor plantar, parestesia, queimação no calcanhar ou na planta do pé, ou sinais neurológicos brandos/inconsistentes, ou ainda naqueles com suspeita de lesões expansivas ou traumas prévios que pudessem causar cicatrizes; o exame revelou anomalias em oito indivíduos. Por fim, a RM foi empregada nos oito pacientes para confirmar a presença das lesões expansivas previamente identificadas pela US e em um paciente com histórico de trauma, revelando as patologias suspeitas. Essas modalidades foram combinadas para redução da incerteza diagnóstica e orientação da indicação cirúrgica.

A intervenção cirúrgica consistiu em uma descompressão aberta do túnel do tarso, realizada sob anestesia regional ou geral pela mesma equipe cirúrgica, com abordagem medial. Os achados intraoperatórios incluíram edema perineural, sinovite e espessamento do retináculo flexor (incisado em sentido longitudinal, com liberação cuidadosa do nervo tibial posterior e seus ramos) em 15 pacientes (100%). Além disso, 8 pacientes (53,3%) tinham lesões ocupando espaço (cistos sinoviais), que foram ressecadas; e 2 pacientes (13,3%) apresentavam fibrose e cicatrizes perineurais, que foram removidas. A hemostasia foi meticulosa e a ferida foi fechada em camadas, sem tensão. Nesta série, nenhum procedimento adjuvante, como neurólise além do nível retinacular ou transferência de tendão, foi realizado.

No período pós-operatório, todos os pacientes seguiram um protocolo padronizado de reabilitação. O tornozelo foi imobilizado em posição neutra por duas semanas e, a seguir, a carga foi gradualmente retomada conforme tolerado. A fisioterapia enfocou exercícios de deslizamento neural, controle do edema perineural e fortalecimento progressivo da musculatura intrínseca e extrínseca. Os pacientes foram acompanhados clinicamente em intervalos regulares até 12 meses após a cirurgia.

O principal desfecho de interesse foi a alteração na pontuação da AOFAS para tornozelo e retropé do início do estudo até 12 meses. Os desfechos secundários incluíram a intensidade da dor, medida pela EVA, e a obtenção da DMCI, definida como uma melhora de ≥ 10 pontos na pontuação da AOFAS para tornozelo e retropé e ≥ 2 pontos na EVA. Esses limiares condizem com os valores de DMCI já publicados para cirurgia do pé e tornozelo, nos quais alterações de 8 a 12 pontos na AOFAS e de 1,5 a 2,0 pontos na EVA demonstraram representar melhoras perceptíveis e clinicamente significativas em pacientes com patologias do retropé ou do túnel do tarso, 8 9 10 bem como a incidência de complicações perioperatórias e a necessidade de nova intervenção cirúrgica.

A análise estatística foi realizada utilizando métodos padrões para dados pareados. As variáveis contínuas foram resumidas como média e desvio-padrão (DP) ou mediana e intervalo interquartil (IIQ), como apropriado. As variáveis categóricas foram expressas em números absolutos e percentagens. O teste t pareado foi escolhido para comparação dos escores pré- e pós-operatórios da AOFAS e da EVA, pois os mesmos indivíduos foram avaliados em dois momentos diferentes, e esse teste avalia as alterações médias em desfechos contínuos.

Embora o tamanho da amostra fosse modesto ( *n* = 15), o teste t é geralmente robusto a pequenos desvios da normalidade, em especial quando a distribuição dos dados é simétrica. Antes da análise, a normalidade dos escores de diferença foi avaliada pelo teste de Shapiro-Wilk e pela inspeção dos gráficos Q-Q; ambos confirmaram uma distribuição aproximadamente normal, corroborando o uso de testes paramétricos. Não obstante, uma análise de sensibilidade utilizando o teste não paramétrico de Wilcoxon para postos sinalizados apresentou níveis de significância comparáveis, confirmando a robustez dos resultados. Os tamanhos do efeito foram calculados utilizando o d de Cohen para quantificar a magnitude da mudança.

As taxas de obtenção de DMCI foram calculadas como proporções. Para explorar os fatores preditivos do desfecho funcional, foi realizada uma análise de covariância (ANCOVA) com o escore AOFAS pós-operatório como variável dependente e o escore AOFAS basal, a idade do paciente, a duração dos sintomas e a positividade à EMG como covariáveis. Um valor de *p* bicaudal < 0,05 foi considerado estatisticamente significativo.

## Resultados

Um total de 15 pacientes consecutivos preencheram os critérios de elegibilidade e foram submetidos à análise após completarem um período de acompanhamento de 12 meses. Suas características basais estão resumidas na Tabela 1 .

**Tabela 1:** Características basais da coorte do estudo

Variável	Valor (n = 15)
Idade, anos (média ± DP)	50,4 ± 15,6
Sexo feminino, n (%)	8 (53,3)
Lateralidade: Esquerda / Direita, n	7/8
Duração dos sintomas, meses (média ± DP)	14,7 ± 7,2
EMG positiva, n (%)	10 (66,7)
RM positiva, n (%)	9 (60,0)
US positiva, n (%)	8 (53,3)

**Abreviações:** DP, desvio-padrão; EMG, eletromiografia; RM, ressonância magnética; US, ultrassonografia.

A avaliação pós-operatória revelou melhoras estatística e clinicamente significativas nos desfechos de função e dor. A pontuação média da AOFAS para tornozelo e retropé aumentou de 36,6 ± 7,1 no pré-operatório para 78,1 ± 19,9 no acompanhamento de 12 meses, correspondendo a um ganho médio de 41,5 ± 18,4 pontos. Essa diferença foi altamente significativa (t = 8,74, *p* < 0,001) e associada a um grande tamanho de efeito (d de Cohen = 2,26), corroborando a robustez do benefício funcional observado. A intensidade da dor, avaliada pela EVA, diminuiu de 7,0 ± 0,6 no início do estudo para 3,4 ± 1,3 na última consulta de acompanhamento, refletindo uma redução média de −3,6 ± 1,0 pontos (t = −14,43; *p* < 0,001; d de Cohen = −3,72). A proporção de pacientes que atingiram a DMCI foi de 80,0% para a AOFAS e 100,0% para a EVA, indicando que as melhoras foram não apenas estatisticamente significativas, mas também clinicamente relevantes para a maioria dos indivíduos. Os resultados detalhados são apresentados na Tabela 2 .

**Tabela 2:** Desfechos funcionais após a descompressão cirúrgica

Desfecho	Pré-operatório (média ± DP)	Pós-operatório 12 meses (média ± DP)	Diferença média (±DP)	Valor de *p*	DMCI (%)
Escore AOFAS	36,6 ± 7,1	78,1 ± 19,9	41,5 ± 18,4	< 0,001	80,0
EVA para dor	7,0 ± 0,6	3,4 ± 1,3	−3,6 ± 1,0	< 0,001	100,0

**Abreviações:** AOFAS, escore da American Orthopaedic Foot and Ankle Society; DP, desvio-padrão; DMCI, diferença mínima clinicamente importante; EVA, escala visual analógica.

A investigação de possíveis fatores preditivos de recuperação funcional foi realizada por meio de um modelo de ANCOVA utilizando o escore AOFAS pós-operatório como variável dependente, com ajuste segundo o escore basal, a idade, a duração dos sintomas e os resultados da EMG. Embora escores basais mais altos e maior duração dos sintomas tenham tendido a influenciar os desfechos resultados funcionais, nenhuma das covariáveis atingiu significância estatística nesta amostra. Este achado pode refletir o tamanho modesto da coorte e as limitações associadas ao poder estatístico. Os resultados do modelo ANCOVA são apresentados na Tabela 3 .

**Tabela 3:** Modelo ANCOVA dos fatores preditivos da pontuação pós-operatória da AOFAS ( *n* = 15)

Covariáveis	Coeficiente β	Valor de *p*
AOFAS basal	1,08	0,159
Idade	0,33	0,465
Duração dos sintomas (meses)	−1,22	0,152
Positividade à EMG	−11,83	0,384

**Abreviações:** AOFAS, escore da American Orthopaedic Foot and Ankle Society; EMG, eletromiografia.

**Fig. 1:**
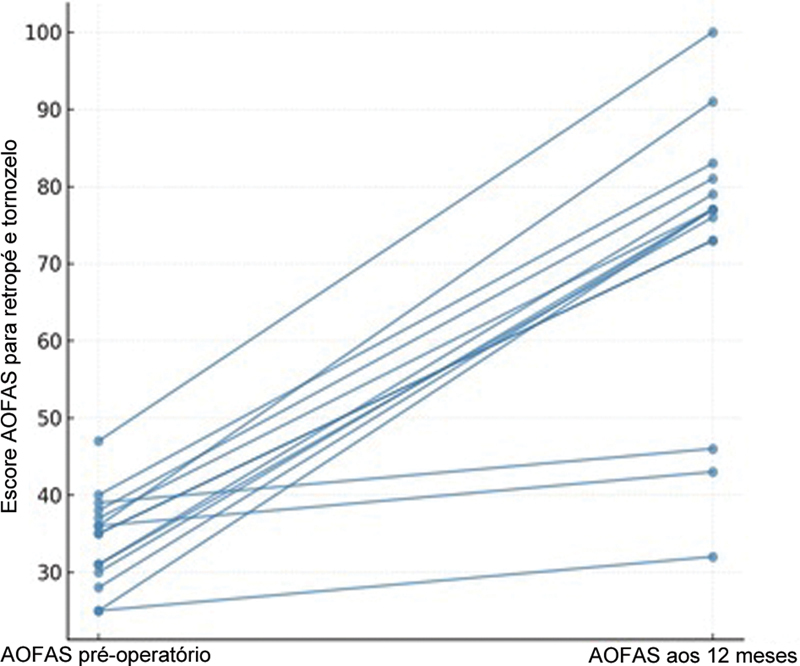
Distribuição dos escores individuais da American Orthopaedic Foot and Ankle Society (AOFAS) antes e depois da descompressão cirúrgica, destacando a melhora consistente observada em toda a coorte.

Foram documentadas três complicações pós-operatórias (20,0%), incluindo dois casos de parestesia transitória e um problema superficial na ferida. Todos foram tratados de forma conservadora e resolvidos sem sequelas. Este é o provável motivo de a pontuação AOFAS pós-operatório ser inferior a 50. É importante ressaltar que nenhum paciente necessitou de nova intervenção cirúrgica durante o período de acompanhamento ( Fig. 1 ).

## Discussão

Nossos achados condizem com séries anteriores relatadas na literatura, em que as taxas de sucesso após a liberação cirúrgica variam entre 70 e 90%. Esses estudos, conduzidos em populações heterogêneas com protocolos diagnósticos e técnicas cirúrgicas diferentes, geralmente demonstraram melhoras significativas tanto na dor quanto nos desfechos funcionais. A magnitude do benefício observado na coorte atual está alinhada ao limite superior dos relatos já publicados, validando ainda mais a eficácia da descompressão quando indicada de maneira apropriada. A incidência relativamente baixa de complicações e a ausência de procedimentos de revisão nesta série também são favoravelmente comparáveis às evidências existentes que, de modo geral, citam problemas menores na ferida ou sintomas neurológicos transitórios como os eventos adversos mais comuns. 11 12 13

Uma característica distintiva deste estudo é a integração sistemática de modalidades diagnósticas auxiliares na avaliação pré-operatória e sua subsequente análise em relação aos desfechos. Os resultados da EMG, RM e US foram variáveis em toda a coorte, refletindo a heterogeneidade e a sensibilidade imperfeita de cada ferramenta. A modelagem exploratória utilizando ANCOVA, embora com poder estatístico limitado devido ao tamanho da amostra, sugeriu que o escore AOFAS basal e a duração dos sintomas podem influenciar a recuperação pós-operatória. Essa tendência pode ser interpretada como um marcador de acometimento neuropático mais avançado ou crônico, em que as alterações estruturais e fisiológicas são menos reversíveis mesmo após a descompressão adequada. Tais observações são clinicamente relevantes, pois destacam a importância de não adiar o encaminhamento cirúrgico em caso de insucesso das medidas conservadoras e persistência dos sintomas por um período razoável. 14 15 16

A pontuação média pós-operatória da AOFAS de 78,1 e a da EVA de 3,4 observadas em nossa coorte indicam uma melhora funcional e sintomática substancial, condizente com uma recuperação clinicamente significativa após a descompressão. Esses resultados estão dentro da faixa relatada em séries anteriores, em que as pontuações pós-operatórias da AOFAS tenderam a variar entre 70 e 85 e da EVA entre 2 e 4, sendo associadas a resultados bons a excelentes. Lalevée et al. 12 , por exemplo, relataram que a pontuação média pós-operatória da AOFAS foi de 80,6 após a liberação aberta do túnel do tarso, enquanto Rungprai et al. 4 observaram ganhos semelhantes independentemente do estado eletrodiagnóstico pré-operatório. Da mesma forma, Iborra et al. 2 e Sun et al. 11 descreveram escores pós-operatória de dor na EVA em torno de 3, correlacionados à alta satisfação do paciente e ao retorno às atividades diárias. Esses achados sugerem que a recuperação funcional alcançada nesta série atende aos parâmetros estabelecidos de sucesso clínico da descompressão do túnel do tarso. Sintomas residuais brandos ou melhoras incompletas em alguns pacientes provavelmente refletem a cronicidade da compressão nervosa ou alterações neuropáticas preexistentes, fenômenos também relatados na literatura. 8 9 10 16 17 18

Do ponto de vista clínico, esses resultados enfatizam duas implicações importantes. Primeiro, a intervenção cirúrgica rápida pode maximizar a recuperação funcional, prevenindo danos neurais irreversíveis associados à compressão prolongada. Segundo, o uso criterioso de exames diagnósticos deve ser adaptado ao contexto clínico: a EMG continua sendo valiosa para documentar o comprometimento neurofisiológico; a RM é particularmente útil para excluir causas secundárias, como lesões ganglionares ou expansivas; e a US oferece uma avaliação dinâmica que pode complementar a imagem estática. A combinação dessas modalidades, interpretadas no contexto de um exame clínico minucioso, permite uma seleção mais precisa dos candidatos à cirurgia. 17 18 19 20

Este estudo apresenta diversas limitações que devem ser discutidas. O tamanho da amostra foi relativamente pequeno, limitando o poder estatístico para detecção de fatores preditivos sutis de desfecho e impedindo uma análise multivariada robusta. O desenho retrospectivo introduz a possibilidade de viés de seleção e depende da completude dos prontuários médicos. Além disso, a ausência de um grupo controle recebendo tratamento não cirúrgico ou cirurgia simulada impede a comparação direta da eficácia do procedimento em questão com o tratamento conservador. O acompanhamento foi limitado a um mínimo de 12 meses, sendo necessários dados em longo prazo para determinar a durabilidade do benefício clínico. 21 22 23

## Conclusão

A descompressão cirúrgica é um tratamento seguro e eficaz para a STT refratária ao tratamento conservador, proporcionando melhoras significativas na dor e na função, com poucas complicações. A intervenção precoce tende a melhorar os desfechos, mas estudos prospectivos de maior porte são necessários para confirmar os fatores prognósticos e esclarecer o valor diagnóstico da EMG, RM e US.
